# Bioinformatic analysis and validation of candidate genes in the eutopic endometrium reveal differential expressions in diffuse adenomyosis, endometrioma, and their co-existence

**DOI:** 10.1186/s40001-025-03412-7

**Published:** 2025-11-21

**Authors:** Imon Mitra, Bishnupriya Saha, Sanjukta Dasgupta, Ankit Kumar Sahu, Subhash Chandra Halder, Sunita Sharma, Ratna Chattopadhyay, Pratip Chakraborty, Koel Chaudhury

**Affiliations:** 1 School of Medical Science and Technology, Indian Institute of Technology Kharagpur, Kharagpur, West Bengal 721302 India; 2 Department of Biotechnology, Center for Multidisciplinary Research & Innovations, Brainware University, Kolkata, West Bengal 700125 India; 3 Department of Bioscience and Biotechnology, Indian Institute of Technology Kharagpur, Kharagpur, West Bengal, 721302 India; 4 Srijoni Healing Home Pvt. Ltd., Kolkata, West Bengal 700010 India; 5 Institute of Reproductive Medicine, Kolkata, West Bengal 700106 India; 6 Cradle Fertility Centre, Kolkata, West Bengal 700104 India

**Keywords:** Adenomyosis, Endometriosis, Co-existent adenomyosis–endometriosis, ECM remodeling, Hub-genes, MMP7, MMP11, MMP9

## Abstract

**Background:**

Adenomyosis and endometriosis are hormone-dependent benign gynecological disorders with overlapping features suggesting that they may share a common origin despite being considered distinct entities. This study compares the expression of candidate genes in the eutopic endometrium of diffuse adenomyosis, ovarian endometriosis, co-existent adenomyosis–endometriosis, and controls.

**Methods:**

Publicly available transcriptomic datasets comprising endometrial tissue of women with adenomyosis, endometriosis, and women with healthy endometrium were analyzed, and overlapping differentially expressed genes (DEGs) were determined. Gene ontology and pathway enrichment analyses were performed to determine shared biological processes. A protein–protein interaction (PPI) network was constructed using the overlapping DEGs and the key genes identified. These genes and corresponding proteins were further validated in the patient population (25 women in each group of diffuse adenomyosis, ovarian endometrioma, co-existent adenomyosis–endometriosis). Thirty women with healthy endometrium having infertile male partners were recruited as controls for comparison purposes. Receiver operating characteristic (ROC) curves were generated for the key genes to evaluate their discriminatory accuracy in classifying isolated adenomyosis. Finally, the gene expressions were correlated with patient clinical characteristics.

**Results:**

23 significant DEGs (log2 fold-change > 1, *p* < 0.05) were found to be common between adenomyosis and endometriosis datasets, with serine-type endopeptidase activity emerging as the most enriched molecular function. PPI network analysis identified *MMP7*, *MMP11*, *IGFBP5*, *SERPINA1*, *THBS1* as the hub genes. In addition, *MMP9* and *TIMP1* exhibited a strong association with the hub gene network. Experimental validation showed altered expression in adenomyosis as compared to controls and other disease groups. MMP9 and MMP7 showed strong discrimination for adenomyosis vs. endometriosis [area under the curve (AUC) = 0.93] and co-existent cases (AUC = 0.97), respectively. The expression of most of the genes in the co-existent group did not align with adenomyosis or endometriosis. *MMP7* expression positively correlated with uterine volume in adenomyosis; *MMP11* could be negatively associated with myometrial wall thickness ratio.

**Conclusions:**

Distinct expression profiles were observed in diffuse adenomyosis versus ovarian endometriosis and co-existent phenotype. Expression of key genes indicated enhanced ECM remodeling in adenomyosis, with MMP7, MMP9, and MMP11 emerging as potential discriminatory markers. The divergent expressions in the co-existent phenotype suggest distinct molecular mechanisms that merit further study.

**Supplementary Information:**

The online version contains supplementary material available at 10.1186/s40001-025-03412-7.

## Introduction

Endometriosis, characterized by the presence of endometrial tissue outside the uterine cavity and affecting approximately 10% of reproductive-age women worldwide, has received considerable research attention over the past few decades in terms of diagnostic challenges, pathogenesis, reproductive outcomes, and novel treatment strategies [1, 2]. Adenomyosis, a similar gynecological condition, is defined by the invasion of the endometrial tissue into the muscular wall (myometrium) of the uterus, affecting up to 20% of women with subfertility [3]**.** Despite both disorders sharing common pathogenic features such as pelvic pain, irregular or heavy menstrual bleeding, and infertility, there is a paucity of longitudinal data on adenomyosis, its progression, as well as its subtype-specific manifestations [4]. Compared to endometriosis, adenomyosis remains relatively less explored at the molecular level, particularly in the areas of comprehensive transcriptomic and proteomic profiling, genome-wide epigenetic regulation, multi-omics integration, and development of non-invasive biomarkers. Only limited studies have investigated its molecular landscape [5–8], underscoring the need for large-scale, subtype-specific analyses.

Adenomyosis is known to impact reproductive and obstetric outcomes more severely than endometriosis, with reduced pregnancy and live birth rates [9, 10]. Both conditions present with multiple subtypes, and heterogeneity is further compounded by varying lesion size and location, extent of infiltration, presence or absence of adhesions, and junctional zone involvement [11]. In clinical practice, the co-existence of endometriosis with adenomyosis is frequently witnessed [12, 13], though the incidence is not well established. A recent study reports sonographic differences in the co-existent adenomyosis–endometriosis group as compared to isolated adenomyosis [14]. Also, significantly reduced pregnancy rates have been observed in the co-existing group when compared to those with endometriosis alone [15]. Our earlier studies are also in agreement with these observations [10], which underlines the need to determine whether the pathophysiology of the co-existent group aligns more closely with adenomyosis or endometriosis.

With advancement in high-throughput next-generation sequencing (NGS), transcriptomics has become a powerful approach for uncovering disease-associated molecular signatures. Initial transcriptomic studies on eutopic endometrium in adenomyosis identified an association between differentially expressed genes (DEGs) and apoptosis, steroid hormone signaling, and extracellular matrix (ECM) remodeling pathways. The authors conclude that a less rigid ECM in the eutopic endometrium may facilitate enhanced migration and invasion of the endometrial cells, potentially contributing to the pathogenesis of adenomyosis [16]. Another similar study established a link between the DEGs and IL6 and MAPK pathways, suggesting a disturbance in the endometrial microenvironment of adenomyosis, as evidenced by increased cellular proliferation and reduced apoptosis [17].

A recent study involving RNA-sequencing of patient-derived endometrial organoids in adenomyosis identified dysregulated molecular mechanisms related to implantation failure and pregnancy disorders as compared to endometrial organoids developed from healthy donors without any disease [18]. Transcriptomic profiling in endometriosis has attracted considerable attention from researchers in recent years. The dysregulated genes expressed in endometriosis were found to be involved in inflammatory pathways [19], epithelial-to-mesenchymal transition (EMT) [20], complement activation, and coagulation [21] pathways. Only two studies exist so far where the expression of DEGs has been compared between adenomyosis and endometriosis. Prasnikar et al. 2020 performed the first integrated transcriptomic analysis and identified six putative genes downregulated in adenomyosis as compared with endometriosis and women without the presence of any uterine or ovarian conditions. Another recent study has shown significant differences in RNA expression levels on comparing focal adenomyosis with deep-infiltrating endometriosis [22, 23]. Additionally, *KRAS* mutations were reported to be more frequent in the endometrium of patients with co-existing adenomyosis–endometriosis patients than in adenomyosis alone [24].

Herein, we analyzed Gene Expression Omnibus (GEO) datasets to identify DEGs in women with adenomyosis compared to disease-free healthy subjects. Next, DEGs overlapping between adenomyosis and endometriosis were identified. Gene Ontology (GO) and protein–protein interaction (PPI) network were used to determine the biological processes and select hub genes, respectively. Expressions of these hub genes and corresponding proteins were further validated in the eutopic endometrium of women with diffuse adenomyosis and compared with those of ovarian endometriosis and co-existent adenomyosis–endometriosis. Subsequently, the transcriptomic signatures were correlated with the clinical characteristics of patients across different disease conditions.

## Materials and methods

The overall workflow of the study design is illustrated in Supplementary Fig. S1.

### Data sources

The Gene Expression Omnibus (GEO) database (https://www.ncbi.nlm.nih.gov/geo/) was searched using the keywords ‘adenomyosis’, ‘endometriosis’ and co-existent adenomyosis-endometriosis. Two datasets were selected for further analysis. The GSE78851 dataset comprised endometrial samples of women with histologically confirmed adenomyosis (*n* = 3) and healthy eutopic endometrium of normo-ovulatory patients (*n* = 5), generated using Affymetrix Human Gene 1.0 ST Array platform. The GSE7307 dataset included endometrial tissue from women with ovarian endometriosis (*n* = 18) and donors having healthy endometrium (*n* = 18). This data was generated using Affymetrix Human Genome U133 Plus 2.0 Array platform. For both datasets, healthy eutopic endometrial samples were collected from women without adenomyosis, endometriosis, or any uterine/ovarian pathology.

Given that interaction between the endometrial and myometrial compartments is central to disease pathogenesis in adenomyosis, myometrial gene expression data (GSE7307; adenomyosis vs. non-adenomyosis cases, 10 subjects in each group) were also considered. Details of both the datasets are provided under the “Availability of data” section and Supplementary Table S1. No datasets were available for the co-existent group.

### Identification of DEGs

Transcriptomic data analysis was conducted using RStudio/Bioconductor. Raw CEL files (.CEL.gz) were retrieved from the GEO database and normalized using the Robust Multi-array Average (RMA) function of the *affy* package. Differential expression analysis was performed using the *limma* package, and gene annotation was carried out using *getGEO* and *GEOquery*. Genes with adjusted *p*-value (padj) < 0.05 (Benjamini–Hochberg correction) and |log2FC|> 1 were considered significant DEGs. Volcano plots were generated to visualize the DEGs and overlapping genes identified via Venn diagram using Venny (https://bioinfogp.cnb.csic.es/tools/venny/) [25]. R scripts used in the analysis are provided as Supplementary files.

### GO and pathway enrichment analysis

Enrichment analysis was performed for the identified DEGs of each transcriptomic dataset using Enrichr (https://maayanlab.cloud/Enrichr/enrich). Next, to explore the biological significance of the overlapping DEGs consistently expressed across adenomyosis and endometriosis, GO enrichment analysis was conducted across three categories: biological process (BP), molecular function (MF), and cellular component (CC), using both EnrichR and g:Profiler (https://biit.cs.ut.ee/gprofiler/gost). KEGG and Reactome databases were employed to identify significantly enriched pathways. Padj < 0.05 (false discovery rate adjusted, Benjamini–Hochberg correction) was set as the threshold for statistical significance.

### Protein–protein interaction network and hub gene identification

To visualize interactions among shared DEGs of adenomyosis and endometriosis, a PPI network was constructed using the STRING database (https://string-db.org) and Cytoscape software (v3.8.2). STRING integrates curated databases to predict physical and functional protein–protein interactions. Nodes represent proteins and edges denote their interactions. Hub genes, defined as the genes with high connectivity within the PPI network, were identified using four topological algorithms (Degree, Maximal Clique Centrality, Density of Maximum Neighborhood Component, and Maximum Neighborhood Component) via the cytoHubba plugin in Cytoscape. Their co-expression and interaction patterns were further explored using STRING. Functional associations were also retrieved using GeneMANIA (https://genemania.org). Finally, the hub genes were selected for experimental validation in patient-derived endometrial tissues.

### Patient selection for experimental validation

This prospective study was approved by the Institutional Ethics Committee of the Institute of Reproductive Medicine (IRM), Kolkata (IRM/IEC/BNC-IHP-06/2021), India, in accordance with the Helsinki Declaration Amendment of 2013. Written informed consent was obtained from all participants prior to the commencement of the study. A total of 105 patients (26–40 years; body mass index (BMI) ≤ 28 kg/m^2^) reporting at IRM, Kolkata, India from July 2021 to December 2023 for infertility treatment were selected for the study. Eligible participants were stratified into four groups: (a) adenomyosis, *n* = 25; (b) endometriosis, *n* = 25; (c) co-existent adenomyosis–endometriosis, *n* = 25; and (d) controls, *n* = 30. Only diffuse adenomyosis and stage III–IV ovarian endometriosis cases were considered. Age and BMI-matched women with normal hormonal profiles, regular menstrual cycles, and no uterine or ovarian pathology evidenced on two-dimensional transvaginal ultrasound (2D-TVUS), having infertile male partners, were considered as controls for comparison purposes.

Women over 40 years of age, with any of the pre-existing gynaecological conditions such as fibroids, ovarian tumors, polycystic ovary syndrome, hydrosalpinx, uterine anatomical abnormalities, and other pelvic pathological conditions, were excluded. Also, women who had received hormonal treatment during the last three months were not included. Adenomyosis was diagnosed based on the Morphological Uterus Sonographic Assessment (MUSA) criteria [26], which required the presence of at least three of the following on 2D-TVUS: heterogeneous myometrium, globular enlargement of the uterus, asymmetrical thickening of the myometrium (excluding fibroids), myometrial cysts, echogenic subendometrial lines and buds, hyperechogenic islands, fan-shaped shadowing, or irregular/interrupted endo-myometrial junctional zone. Diagnostic laparoscopy was performed in these diffuse adenomyosis cases to exclude the presence of concomitant endometriosis.

Ovarian endometriosis and co-existent adenomyosis–endometriosis were confirmed via laparoscopy, histopathology, and ultrasound detection of unilateral or bilateral endometriomas. Direct and indirect sonographic features of adenomyosis were recorded for both adenomyosis and co-existent groups [27].

All ultrasound evaluations were performed using Voluson™ P8 (GE HealthCare, Austria) by a single experienced sonologist to minimize inter-observer variability. Uterine volume was calculated using the ellipsoid formula (longitudinal diameter × transverse diameter × anteroposterior diameter × 0.532). Myometrial wall thickness was measured in the sagittal plane, and the anterior-to-posterior myometrial wall thickness ratio was calculated to assess asymmetry, with deviations from 1 indicating disproportion [28]. Myometrial wall asymmetry was compared across all groups. The participants were monitored during their subsequent IVF cycle, and reproductive outcomes were recorded. Also, their basal hormonal levels were noted within 5 days of the menstrual cycle.

### Sample collection

At the time of routine hysteroscopic evaluation prior to IVF cycle, eutopic endometrial tissue was collected from the subjects during their proliferative phase using an endometrial suction curette (Probet, Gynetics Medical Products N.V., Lommel, Belgium). The tissue was rinsed in 1X phosphate-buffered saline and immediately divided into two parts: one for protein extraction and the other for isolation of RNA. Both fractions were snap-frozen immediately and stored at –80 °C until further processing.

### Quantitative reverse transcription PCR (qRT-PCR)

Approximately 40–50 mg of endometrial tissue was collected from all subjects (*n* = 20 in each group), and total RNA was extracted using TRIzol™ (Thermo Fisher Scientific, USA). RNA concentration and purity were assessed using a NanoDrop™ 2000 UV spectrophotometer (Thermo Fisher Scientific, USA). Reverse transcription was performed for 30 cycles using the iScript™ cDNA Synthesis Kit (1708890, Bio-Rad, USA) in 20 µL reaction mixture. Next, qRT-PCR was performed using specific primers and amplified with SYBR™ Green Universal Master Mix (43-091-55, Applied Biosystems StepOne^®^) on a Real-Time PCR System (Applied Biosystems). The relative gene expression was quantified using the 2^−ΔΔCt^ method [29], with *GAPDH* as the internal reference/control. ΔCt was calculated as the difference between the Ct values of the target gene and GAPDH, and ΔΔCt was derived by comparing ΔCt values between experimental and control groups. Ct represents the threshold cycle at which fluorescence exceeds the background, indicating exponential amplification. All experiments were performed in triplicate. Primer sequences are listed in Table 1.

**Table 1:** List of primers used in quantitative reverse transcriptase polymerase chain reaction (qRT-PCR)

Name of gene	Sequence	
	Forward primers (5′- 3′), Tm	Reverse primers (5′- 3′), Tm
*MMP7*	GCTCACTTCGATGAGGATGAA, 54.5 °C	AGGAATGTCCCATACCCAAAG, 54.5 °C
*MMP11*	TTGACCCTGTGAAGGTGAAG, 54.6 °C	AAGCCATGGTCAGAGGAAAG, 54.8 °C
*MMP9*	GCTGATGGCTATGCCTACTT, 54.5 °C	AAGTCAGGACCCACGAGA, 54.6 °C
*SERPINA1*	GGTCAACTGGGCATCACTAA, 54.8 °C	GATGGTCAGCACAGCCTTAT, 54.9 °C
*IGFBP5*	TTCCACCCATTCTCCCTTTG, 54.9 °C	GTAGTTCCTGGCTCAGTCTTT, 54.2 °C
*THBS1*	AAGCACACGCAACTCTCA, 54.6 °C	CTCCTCCCTCATCCACATTTAC, 54.8 °C
*TIMP1*	TCCGTCCACAAGCAATGAG, 55.5 °C	GGCTTCACCAAGACCTACAC, 55.2 °C
*GAPDH*	CCCTTCATTGACCTCAACTACA, 57.4 °C	ATGACAAGCTTCCCGTTCTC, 61.3 °C

Tm melting temperature, *MMP7* matrix metalloproteinase 7, *MMP11* matrix metalloproteinase 11, *SERPINA1* serine protease inhibitor, clade A, member 1; *MMP9* matrix metalloproteinase 9, *IGFBP5* insulin-like growth factor binding protein 5, *THBS1* thrombospondin 1, *TIMP1* tissue inhibitor of metalloproteinase 1, *GAPDH* glyceraldehyde-3-phosphate dehydrogenase

### Western blot

Total protein was extracted from the endometrial tissue using RIPA lysis buffer (#9806, Cell Signaling Technology, USA) supplemented with protease inhibitor cocktail (GE Healthcare; 80-6501-23, Chicago). The total protein concentration was measured using the bicinchoninic acid (BCA) assay (23227, Thermo Fisher Scientific, USA). Equal amounts of protein were separated on a 10% SDS–polyacrylamide gel and transferred onto a nitrocellulose membrane using a semi-dry transfer system (TransBlot Turbo, Bio-Rad, USA) at 20 V, 1.5 mA for 12 min. Membranes were blocked with 5% non-fat dry milk in TBST (Tris-buffered saline with 0.1% Tween 20; Bio-Rad; #1706531) for 1 h at room temperature and incubated overnight at 4 °C with primary antibodies against MMP7, MMP9, MMP11, SERPINA1, THBS1, TIMP1, and IGFBP5 (1:1000 dilution, Supplementary Table S2). After washing, the membranes were incubated with HRP-conjugated anti-rabbit or anti-mouse secondary antibodies (7074 and 7076, Cell Signaling Technology, USA; 1:3000 dilution) for 3 h at room temperature. After three TBST washes (5 min each), the membranes were developed using ECL substrate (1705060, Bio-Rad, USA) and visualized with a ChemiDoc imaging system (Bio-Rad, USA). Beta-actin (10494-1-AP, ABclonal Technology, USA) was used as an internal control. Protein band intensities were analyzed using ImageJ 1.48v software (NIH, USA), and results were normalized to beta-actin. Each experiment was performed three times.

### Statistical analysis

Clinical variables were assessed using the Kruskal–Wallis test for comparisons across multiple groups and the Mann–Whitney *U* test for pairwise comparisons. Normality of distribution was evaluated using the D’Agostino–Pearson omnibus test. Gene and protein expression data were analyzed using one-way analysis of variance (ANOVA) followed by Tukey’s post hoc test for multiple comparisons. Receiver operating characteristic (ROC) curves were generated to assess the discriminative performance of the hub genes and proteins between adenomyosis vs. endometriosis and the co-existent group; area under the curve (AUC) values were calculated using the Wilson/Brown method.

Spearman’s rank correlation coefficient was used to analyze the correlation between the expression of hub genes and clinical characteristics. The correlation coefficient (r) > 0.5 and *p* 0.05 was considered statistically significant. All analyses were computed using GraphPad Prism version 9.0 (GraphPad Software, San Diego, CA). Data are expressed as mean ± standard deviation (SD), unless otherwise specified.

## Results

### Identification of DEGs in adenomyosis

**Fig. 1:**
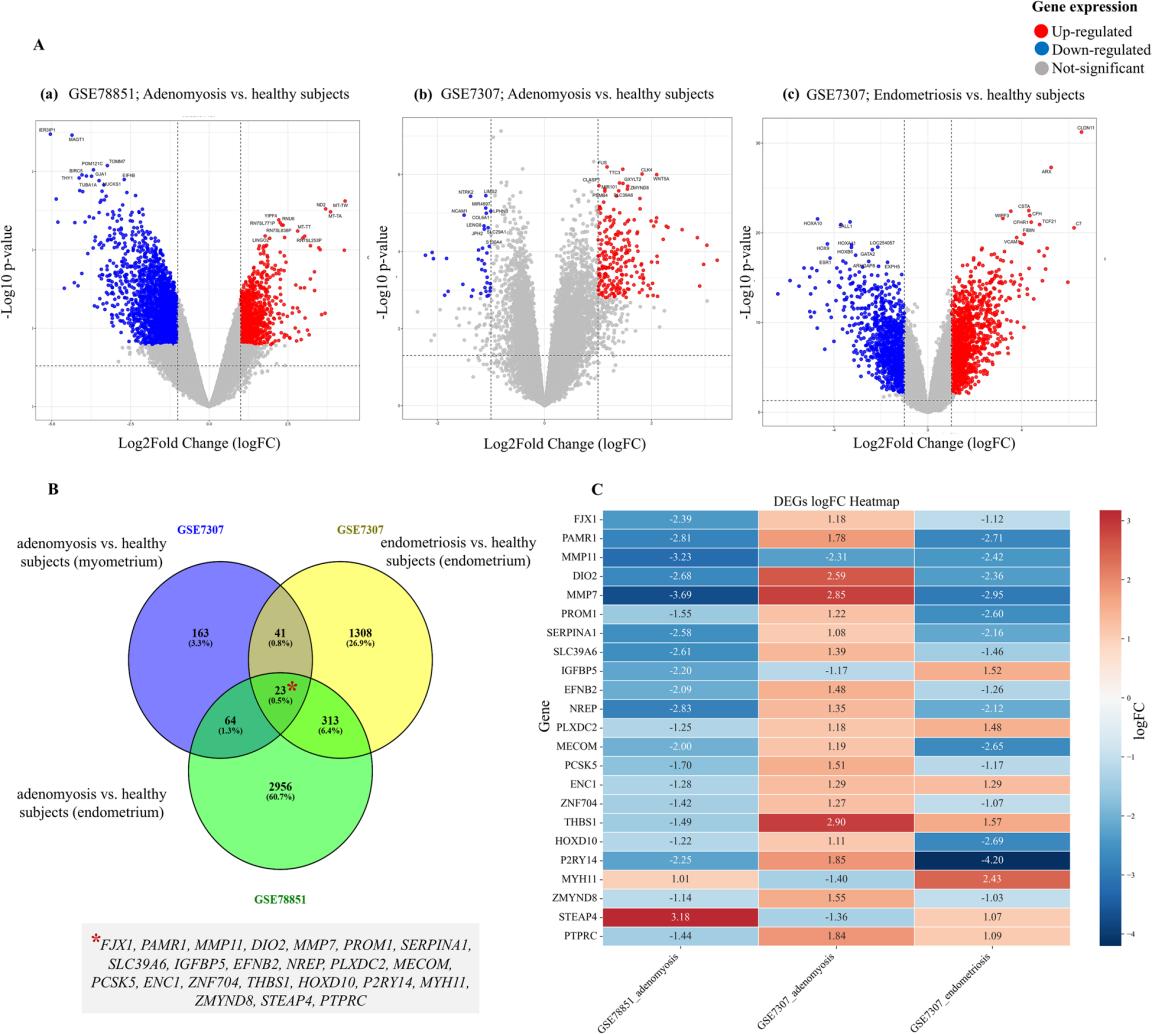
Volcano plots of datasets and Venn diagram of common differentially expressed genes (DEGs). **A** Volcano plots show significantly upregulated and downregulated DEGs in a adenomyosis vs. healthy subjects (GSE78851 dataset), **b** adenomyosis vs. healthy subjects (GSE7307 dataset), **c** endometriosis vs. healthy subjects (GSE7307 dataset). X and Y-axes represent logFC values and –logp-values, respectively. Significantly upregulated (red dots) and downregulated genes (blue dots) with an adjusted *p*-value < 0.05 and |log2FC ≥ ± 1| were identified following Benjamini–Hochberg correction. **B** A total of 23 DEGs common between GSE7307 (adenomyosis vs. healthy subjects; myometrium—blue circle), GSE7307 (endometriosis vs. healthy subjects; endometrium—yellow circle) and GSE78851 (adenomyosis vs. healthy subjects; endometrium—green circle) are shown. Venn diagram is computed by Venny tool version 2.1 (https://bioinfogp.cnb.csic.es/tools/venny/). **C** Log fold change (logFC) heatmap of the common DEGs across the three sets of data

A total of 4257 DEGs were identified in the GSE78851 dataset (eutopic endometrium of adenomyosis vs. disease-free endometrium of normo-ovulatory women), with 1780 DEGs upregulated and 2477 downregulated ( *padj* < 0.05, |log2FC|> 1). Analysis of the second GSE7307 dataset (myometrium of adenomyosis vs. healthy myometrium of donors) identified 455 DEGs, of which 409 were upregulated and 46 downregulated. The same dataset, when analyzed for endometriosis vs. healthy donors, yielded 2560 DEGs, with 1145 of them upregulated and 1415 downregulated. Volcano plots and box plots of the gene-expression data are provided in Fig. 1A and Fig. S2, respectively. Cross-comparative analysis using a Venn diagram identified 23 DEGs common across both the datasets (Fig. 1B). Log2FC of the overlapping DEGs for the datasets is provided in Fig. 1C and Supplementary Table S3.

### GO and pathway enrichment analysis

The complete GO enrichment results for each set of transcriptomic profile data are provided in the Supplementary Files. GO enrichment analysis of the DEGs shared by adenomyosis and endometriosis indicated their involvement in biological processes related to regulation of the transforming growth factor-beta signaling pathway (GO:0017015) and chemotaxis (GO:0050920). Serine–type endopeptidase activity (GO:0004252) emerged to be the most significantly enriched molecular function (padj < 0.05), as evidenced by both the tools Enrichr and gProfiler. Platelet alpha granule lumen (padj < 0.05) and intracellular organelle lumen (padj < 0.05) were the top two enriched cellular components.

**Fig. 2:**
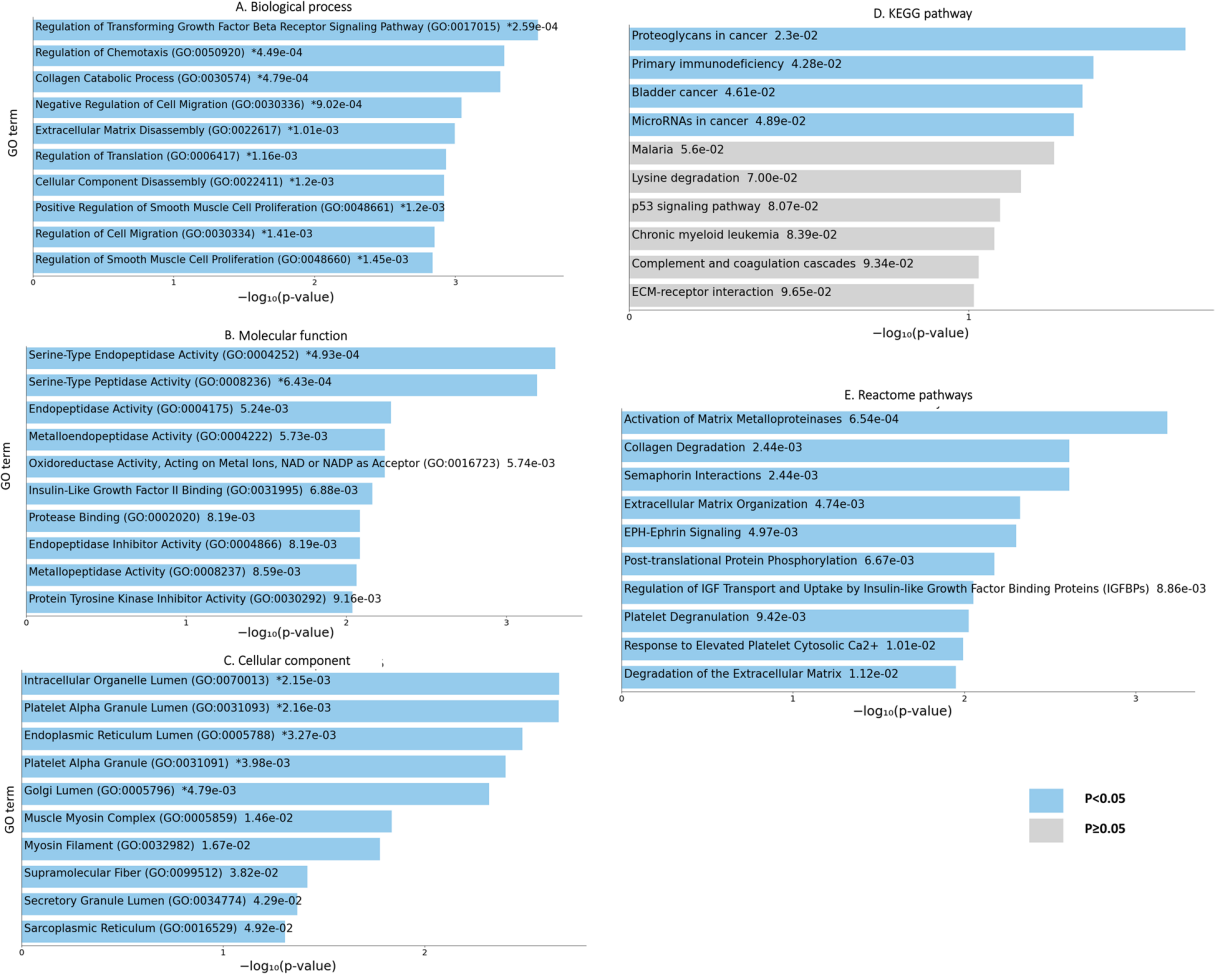
Gene Ontology (GO) enrichment heatmaps associated with the differentially expressed genes (DEGs). The top ten GO terms enriched for the following categories: **A** biological processes, **B** molecular functions, **C** cellular components, **D** Kyoto Encyclopedia of Genes and Genomes (KEGG), and E reactome pathways are shown based on their -log10(*p*-value), with the corresponding* p*-values provided next to each term. Each row represents an enriched GO term, with the length of the bar indicating the level of significance, i.e., the topmost bar (longest one) corresponds to the term with the smallest *p*-value (highest significance). Blue color denotes *p* < 0.05, grey *p* ≥ 0.05

Pathway enrichment analysis highlighted the activation of matrix metalloproteases (MMPs) (RHSA-1592389) and proteoglycans in cancer (hsa05205) as top pathways; however, the values were not significant (padj = 0.06). Enrichment heatmaps representing the top ten GO terms and pathways are shown in Fig. 2. The detailed enrichment analysis results are provided in Supplementary Tables S4 and S5.

### PPI network and identification of hub genes

**Fig. 3:**
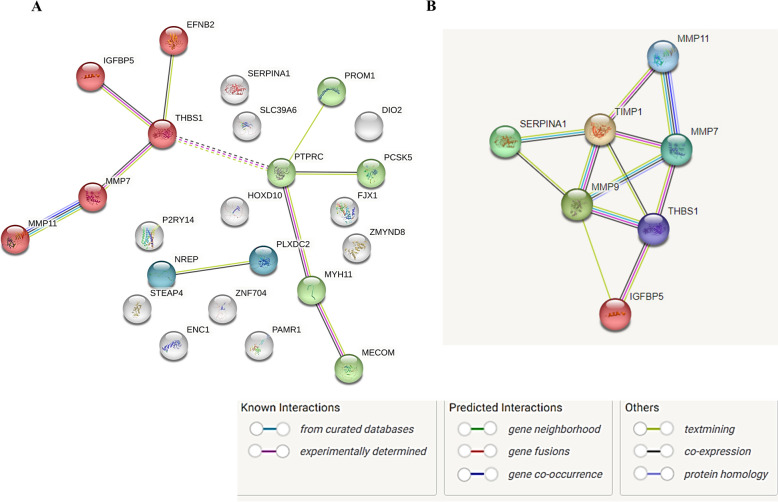
Protein–protein interaction (PPI) networks of differentially expressed genes (DEGs). **A** PPI network of common DEGs between adenomyosis and endometriosis, visualized using STRING (Search Tool for the Retrieval of Interacting Genes Database; http://string-db.org). The network comprises 23 nodes (proteins) and 10 edges (interactions) with significant enrichment (*p* < 0.001). Red nodes indicate genes belonging to the specified cluster from k-means clustering analysis. **B** PPI network of hub genes of interest (7 nodes and 12 edges) exhibited clustering coefficient of 0.76

The network of overlapping DEGs visualized by STRING shows 23 nodes and 10 edges (enrichment p-value < 0.001) (Fig. 3). K-means clustering of these DEGs identified a central module comprising *MMP7*, *MMP11*, *THBS1*, *IGFBP5*, and *EFNB2* genes (Fig. 3A), which, according to STRING database analysis, was functionally linked to extracellular matrix (ECM) remodeling and collagen degradation.

Next, topological analysis using the cytoHubba plugin in Cytoscape identified *MMP7*, *MMP11*, *SERPINA1*, *THBS1*, and *IGFBP5* as candidate hub genes expressed in both adenomyosis and endometriosis (Fig. 3B). These genes formed a tightly connected sub-network of 7 nodes and 12 edges with a local clustering coefficient of 0.76 and significant interaction enrichment (*p* = 2.39 × 10⁻⁹). GO and functional analysis associated with the hub gene network confirmed the presence of few ECM remodeling pathways, with the collagen catabolic process (BP: GO0030574) and ECM disassembly (BP: GO0022617) emerging as the most enriched biological processes (Table 2). Functional annotations associated with the hub genes retrieved via the GeneMania network are provided in Supplementary Table S6. *MMP9* and *TIMP1* with node scores > 0.9 could be identified as additional high-confidence nodes (Supplementary Table S7). The five hub genes were further considered for validation in the patient cohort.

**Table 2:** Gene Ontology (GO) and functional annotations associated with the hub genes

GO category and term	Description	FDR
BP: GO0030574	Collagen catabolic process	0.005
BP: GO0022617	ECM disassembly	0.005
BP: GO0010951	Negative regulation of endopeptidase activity	0.005
BP: GO0048661	Positive regulation of smooth muscle cell proliferation	0.007
MF: GO:004222	Metalloendopeptidase activity	0.03
CC: GO0031012	ECM	< 0.0001
CC: GO0031093	Platelet alpha granule lumen	0.0009

*GO* Gene Ontology, *BP* biological process, *MF* molecular function, *CC* cellular component, *ECM* extracellular matrix, *FDR* false discovery rate, FDR calculated after correcting p-values for multiple testing within each category using the Benjamini–Hochberg procedure

### Clinical characteristics of the validation patient cohort

Clinical characteristics of the patient population enrolled for validation of genes of interest are summarized in Table 3. No significant differences were observed in terms of age, BMI, and duration of infertility across the four groups. Among the hormonal parameters assessed, estradiol (E2) levels differed significantly between adenomyosis and controls (*p* < 0.05); follicle-stimulating hormone (FSH) and luteinizing hormone (LH) levels remained comparable. Representative TVUS images of endometrial tissue are provided in Supplementary Fig. S3. Direct sonographic features of adenomyosis were identified in 34% of the patients with isolated adenomyosis and in 19% of those with co-existent adenomyosis–endometriosis. The indirect features were exhibited by 42% and 75% of the adenomyosis and co-existent group, respectively. The most common ultrasound features observed in > 80% of patients with adenomyosis as well as co-existent adenomyosis–endometriosis were asymmetrical thickening of the myometrial walls and heterogeneous myometrium. More than 80% of the adenomyosis cases and nearly 60% of the combined adenomyosis–endometriosis cohort exhibited interrupted or irregular junctional zone. The presence of myometrial cyst was observed in 56% of the women with isolated adenomyosis. The uterine volume and myometrial wall thickness ratio differed significantly on comparing adenomyosis with controls (*p* < 0.001) and with endometriosis (*p* < 0.001). The clinical pregnancy rate was observed to be 20% and 24% among women with isolated adenomyosis and co-occurring adenomyosis–endometriosis, respectively. The live birth rate was 12% in women with adenomyosis, 28% in endometriosis, and 16% in co-existent adenomyosis–endometriosis cases. A higher incidence of miscarriage was reported in adenomyosis (44%) and co-existent cases (40%) than in patients with endometriosis alone (32%).

**Table 3:** Baseline characteristics of patient cohort

Parameters	Adenomyosis- A (*n* = 25)	Endometriosis- E (*n* = 25)	Co-existent adenomyosis-endometriosis- AE (*n* = 25)	Controls-C (*n* = 30)	*p*-value			*p*-value (4 groups)
					A vs. E	A vs. AE	A vs. C	
Age (years)	32.15 ± 4.92	31.62 ± 5.15	32.22 ± 3.88	32.34 ± 3.89	ns	ns	ns	ns
BMI (kg/m^2^)	25.19 ± 2.83	23.81 ± 2.14	24.25 ± 2.64	23.99 ± 2.39	ns	ns	ns	ns
Duration of infertility (years)	8.00 ± 4.09	6.59 ± 3.64	9.05 ± 4.64	7.62 ± 2.63	ns	ns	ns	ns
E2 (pg/ml)	38.07 ± 11.06	45.09 ± 11.11	35.85 ± 25.14	27.11 ± 14.37	ns	ns	*	*
FSH (mIU/ml)	6.8 ± 2.9	8.69 ± 4.9	7.56 ± 3.28	7.22 ± 2.31	ns	ns	ns	ns
LH (mIU/ml)	5.51 ± 1.53 5.54	6.08 ± 1.44	5.31 ± 2.29	5.06 ± 2.89	ns	ns	ns	ns
Uterine volume (cc)	92.25 ± 38.49	40.54 ± 13.47	53.06 ± 24.18	31.26 ± 8.59	***	ns	***	****
Irregular/interrupted junctional zone (% of patients)	85%	–	60%	–	–	–	–	–
Myometrial wall thickness ratio	0.60 ± 0.26	0.96 ± 0.16	0.79 ± 0.34	1.04 ± 0.09	**	ns	***	***
Dysmenorrhea, n (%)	19 (76%)	21 (84%)	18 (72%)	–	–	–	–	–
History of miscarriage, n (%)	11 (44%)	8 (32%)	10 (40%)	–				–
Pregnancy rate, n (%)	5 (20%)	10 (40%)	6 (24%)	12 (40%)				
Live birth rate (%)	3 (12%)	7 (28%)	4 (16%)	9 (36%)				

Data are represented as mean ± SD, *p value < 0.05; **p value < 0.01; ***p value < 0.001****p < 0.0001. The four groups are compared using Kruskal–Wallis non-parametric test and Mann–Whitney *U*-test is used to compare between two groups. *BMI* body mass index, *E2* estradiol, *FSH* follicle-stimulating hormone, *LH* luteinizsing hormone, *ns* non-significant

### Candidate genes and proteins assessment in eutopic endometrium of patient population

**Fig. 4:**
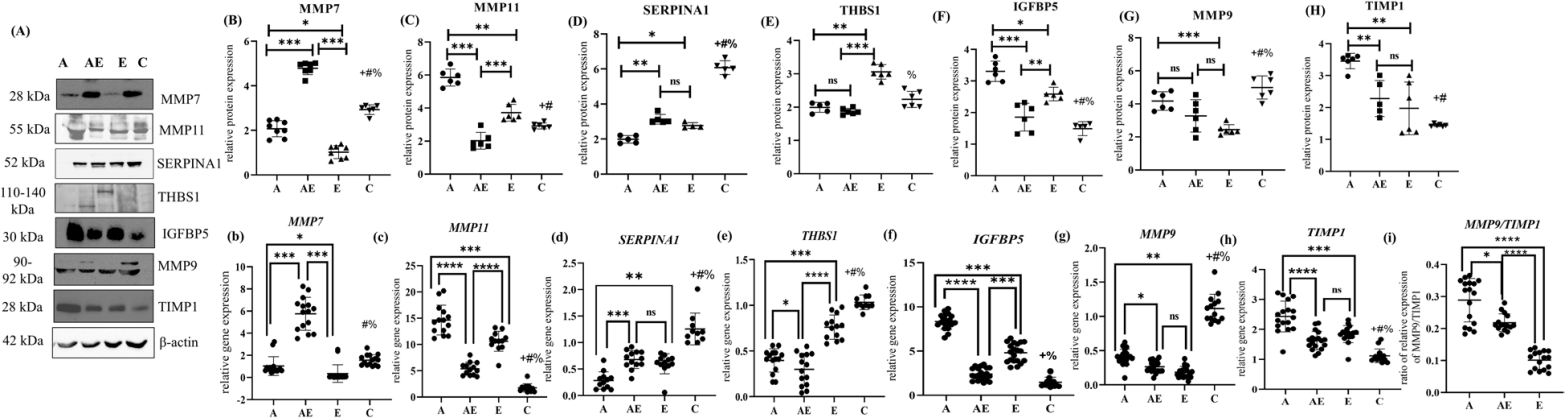
Gene and protein expressions of the candidate molecules. **A** Representative western blot images of proteins of interest and beta-actin with their corresponding molecular weights. The four patient groups are represented as adenomyosis-A, co-existent adenomyosis–endometriosis-AE, endometriosis-E, and controls-C (**B**) to (**H**). The dot plots represent band intensity values of the proteins MMP7, MMP11, SERPINA1, THBS1, IGFBP5, MMP9 and TIMP1, respectively, normalized with the band intensity of beta-actin (*n* = 4) (**b**) to (**h**). The dot plots show the mRNA expression values of the corresponding genes of interest *MMP7, MMP11, SERPINA1, THBS1, IGFBP5, MMP9* and *TIMP1. GAPDH* was used as the reference gene for quantitative reverse transcription PCR (qRT-PCR) analysis [*n* = 20 in each group] (**i**) *MMP9*/*TIMP1* ratio. *P*-values were determined and adjusted following comparison of the four groups using one-way ANOVA with Tukey’s multiple comparison post hoc test. Horizontal lines in the plots denote the mean, and error bars indicate the standard deviation (SD). ****p_adj_ < 0.0001, ***p_adj_ < 0.001, **p_adj_ < 0.01, and *p_adj_ < 0.05 denote statistical significance within the groups; + *p*_adj_ < 0.05: adenomyosis vs. controls, # *p*_adj_ < 0.05: co-existent adenomyosis–endometriosis vs. controls and % *p*_adj_ < 0.05: endometriosis vs. controls. ns: non-significant

On comparing adenomyosis patients with controls, significant differences were observed in the expression of MMP9, MMP11, SERPINA1, IGFBP5, and TIMP1 (*p* < 0.05); however, MMP7 and THBS1 remained comparable. Comparison of gene and protein expression levels among adenomyosis, endometriosis, and co-existent adenomyosis–endometriosis revealed distinct expression patterns, prompting further investigation of within-group differences. MMP7, MMP9, MMP11, and TIMP1 were observed to be significantly upregulated in women with adenomyosis as compared to endometriosis. While MMP7 exhibited the highest expression in the co-existent group (*p* < 0.0001), MMP11 and IGFBP5 showed maximum expression in women with adenomyosis (*p* < 0.0001). However, SERPINA1 and THBS1 were found to be significantly downregulated in these women relative to endometriosis. Further, MMP9 was found to be downregulated and TIMP1 upregulated in all the three diseased groups as compared to controls. Given the importance of MMP9/TIMP1 balance in ECM turnover, the *MMP9*/*TIMP1* ratio was assessed. Adenomyosis exhibited more than threefold and onefold increases as compared to endometriosis and co-existent adenomyosis–endometriosis, respectively. All gene and protein expression levels are shown in Fig. 4 (full blots provided in Supplementary Fig. S4).

**Fig. 5:**
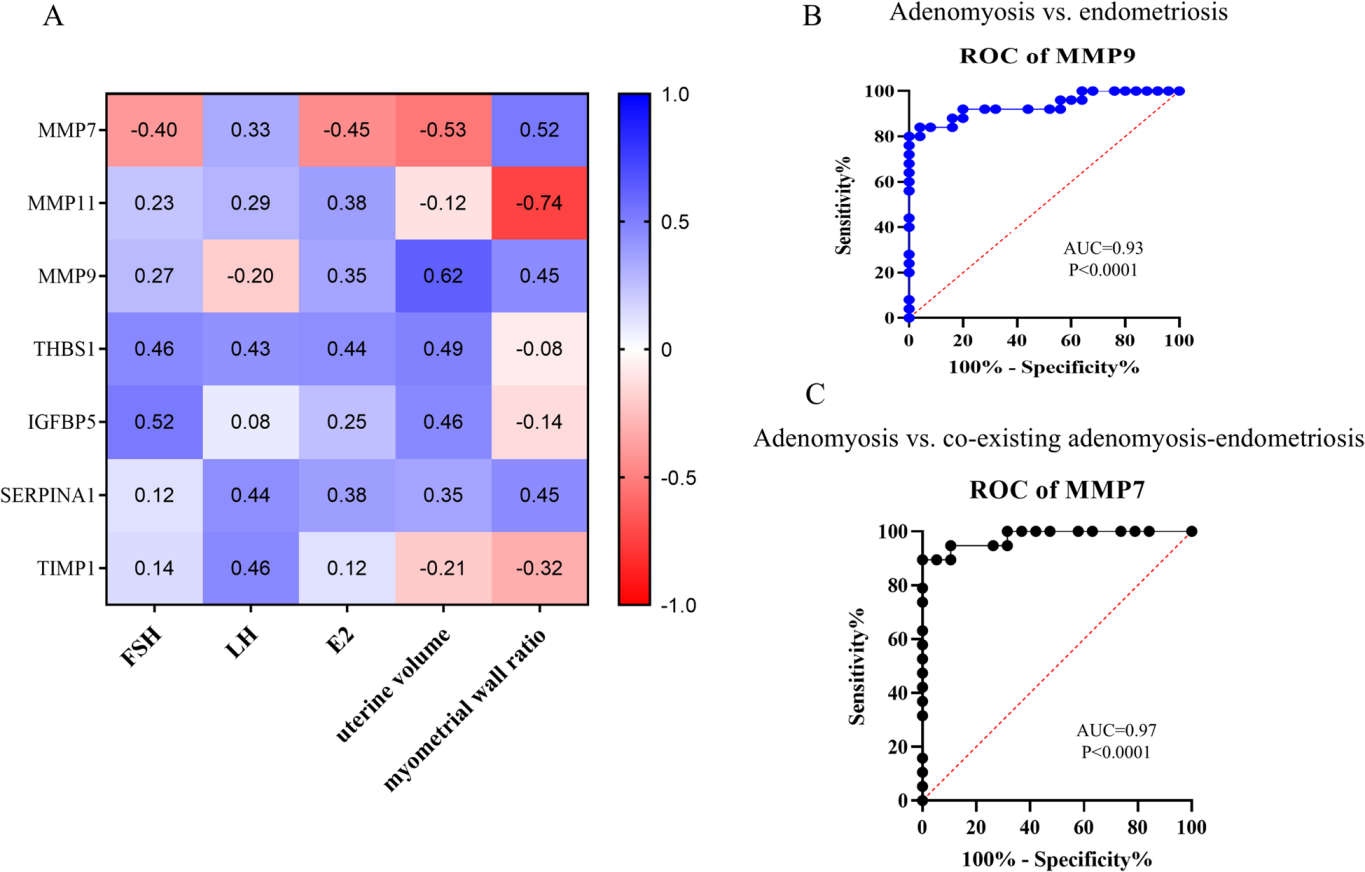
Heatmap and receiver-operating characteristic (ROC) curves of candidate genes with the highest area-under-the-curve (AUC). **A** Correlation heatmap of clinical characteristics of adenomyosis and hub genes’ expressions. Each matrix cell represents the Spearman correlation coefficient r-value. The color gradient reflects the r-values, ranging from red (lowest value of − 1), through white (baseline value 0), to blue (highest value + 1). r-value > 0.5 and *p* < 0.05 denote significance. The scale bar indicates the range of r-values. **B** ROC curve of MMP9 exhibits the highest discriminatory ability (AUC = 0.93) in effectively differentiating between adenomyosis and endometriosis. **C** MMP7 demonstrates the highest discriminative potential (AUC = 0.97) in distinguishing adenomyosis from the co-existent adenomyosis–endometriosis group. The curves are generated by plotting sensitivity% vs. 100%-specificity and thereafter AUC scores calculated. FSH follicle-stimulating hormone, LH luteinizing hormone, E2 estradiol

MMP9 emerged to be the best performance indicator in differentiating between adenomyosis and endometriosis (AUC = 0.93; 95% confidence interval (CI) 0.86–1.00; *p* < 0.0001; Fig. 5B), followed by MMP11 (AUC = 0.82; 95% CI 0.70–0.94, *p* < 0.001). In contrast, on comparing adenomyosis with the co-existent adenomyosis–endometriosis group, MMP7 demonstrated the highest discriminatory power (AUC = 0.97, *p* < 0.0001), indicating its potential as a key marker for distinguishing isolated adenomyosis from overlapping pathology (Fig. 5C; Supplementary Table S8).

### Correlation of genes of interest with clinical parameters

Figure 5 and Supplementary Table S9 present the association between the key genes and clinical characteristics of adenomyosis patients. A negative correlation could be observed between myometrial wall thickness ratio and *MMP11* expression (*r* = − 0.74, *p* < 0.05). Uterine volume correlated positively with *MMP9* (*r* = 0.62, *p* < 0.01), and negatively with *MMP7* (*r* = − 0.53, *p* < 0.05).

## Discussion

Adenomyosis, despite its association with adverse reproductive outcomes, remains less explored than endometriosis. This gap motivated us to apply bioinformatics analyses for the identification of DEGs in the eutopic endometrium of adenomyosis and endometriosis. Further, gene and protein expression levels of the candidate molecules were validated in women with diffuse adenomyosis, ovarian endometriosis, co-existent adenomyosis–endometriosis, and healthy controls. The major genes of interest were also correlated with the clinical characteristics of adenomyosis patients.

Bioinformatics analyses demonstrated serine-type endopeptidase activity and collagen binding activity as leading molecular functions associated with the common DEGs, implicating proteolytic and fibrotic components as major players in the pathophysiology of adenomyosis as well as endometriosis. These findings are consistent with earlier reports highlighting MMP-driven ECM remodeling in the eutopic endometrium of both diseases [30, 31]. Enrichment in the lumen of platelet alpha granules and intracellular organelle lumen underscores the involvement of platelets in tissue repair and ECM remodeling via localized protease release and signaling [32]. The identified DEGs were linked to ECM remodeling and collagen degradation based on STRING network analysis. This is consistent with the known functions of the component genes. MMP7 and MMP11 are well-characterized proteases that degrade collagen IV, fibronectin, and other ECM substrates, thereby contributing to ECM turnover [33]. THBS1 modulates cell–matrix interactions and activates latent TGF-β, promoting ECM deposition and fibrosis [34]. IGFBP5 induces collagen production through multiple signaling pathways, underscoring its role in fibrogenesis [35, 36]. EFNB2 is involved in vascular remodeling, cell adhesion, and structural organization, contributing to ECM dynamics in pathological states [37]**.**

The seven candidate genes were found to be differentially expressed not only between adenomyosis and controls but also across various diseased groups. Out of these, significant overexpression of MMP7, MMP9, MMP11, IGFBP5, and TIMP1 was observed at both gene and protein levels in adenomyosis as compared to endometriosis. These observations may be attributed to junctional zone involvement in adenomyosis and the co-existent adenomyosis–endometriosis group of patients.

We found the expression of MMP7 to be significantly less in the eutopic endometrium of adenomyosis cases as compared to controls. Our results are consistent with the findings of Herndon et al., where downregulation of MMP7 is reported [16]. Interestingly, MMP7 expression was observed to be the highest in the co-existent adenomyosis–endometriosis group, suggesting a synergistic effect on ECM degradation in the presence of dual pathology, potentially driving more aggressive tissue remodeling. We also found the expression of *MMP7* to be inversely related to the uterine volume of adenomyosis patients. It is important to mention that MMP7 outperformed the other genes in distinguishing adenomyosis from co-existent adenomyosis–endometriosis (AUC = 0.97).

MMP11 expression was observed to be the highest in the eutopic endometrium of adenomyosis patients, which could be due to increased proteolytic activity in the endo-myometrial junction of these women. Overexpression of MMP11 is reported in the literature for both adenomyosis [38] and endometriosis [39]. Additionally, *MMP11* inversely correlated with the myometrial wall thickness ratio in adenomyosis. Our observations suggest the involvement of MMP11 in repair mechanisms associated with myometrial auto-traumatization and aggressive behavior of endometrial cells in the eutopic endometrium [40]. The expression of IGFBP5, an estrogen-regulated molecule and substrate of MMP11, was also the highest in adenomyosis and lowest in the co-existent group. The role of IGFBP5 in promoting collagen synthesis and cell proliferation is well established [41].

We found MMP9 levels to be significantly less in adenomyosis, endometriosis, and co-existent adenomyosis–endometriosis groups as compared to controls. This is likely due to increased collagen deposition and a pro-fibrotic environment in the eutopic endometrium of all three diseased conditions. Further, MMP9 expression was the lowest in the endometriosis group of patients. Inconsistent findings are documented regarding the expression of MMP9; while Zhai and co-workers reported it to be substantially elevated in adenomyosis [42], both upregulation and downregulation of the molecule are documented in endometriosis [43, 44]. Aberrant MMP9 regulation is implicated in irregular uterine expansion and complicated pregnancies [45]. A positive correlation between uterine volume and *MMP9* expression in adenomyosis was noticed. Conversely, the endogenous inhibitor of MMP9, viz. TIMP1 exhibited upregulation in adenomyosis as compared to controls as well as endometriosis. This is in agreement with earlier transcriptomic studies where mRNA levels of *TIMP1* showed higher expression in decidualized endometrial stromal cells of adenomyosis [42]. It is well established that the *MMP9*/*TIMP1* imbalance alters the endometrial vasculature and ECM turnover considerably. The ratio was significantly higher in adenomyosis than in the other two disease groups, indicating aggressive ECM proteolysis in this condition. Further, MMP9 demonstrated strong discriminatory potential in the classification of adenomyosis and endometriosis (AUC = 0.93).

Downregulation of the serine protease inhibitor, SERPINA1, in all three disease conditions suggests impaired protease activity. The expression was minimal in adenomyosis, indicating the availability of a more permissive endometrial matrix environment in these women. THBS1 is implicated in cell–matrix interaction and exhibits anti-angiogenic properties by inhibiting endothelial cell proliferation in the endometrium. It is known to be associated with uterine inflammatory myofibroblastic tumors [46]. We observed the gene expression levels of *THBS1* to be significantly lower in all three disease groups, with the least expression in co-existent adenomyosis–endometriosis cases. On the contrary, the protein expression was seen to be the highest in endometriosis. There exists only one study so far where increased *THBS1* gene expression in adenomyosis is reported [38]. A definitive explanation for the observed expression pattern is not possible due to the limited number of studies available.

There are several limitations in our study. First, the limited sample size associated with the GEO datasets and the validation patient cohort require the study to be extended to a larger patient population. Second, the exact staging of adenomyosis could not be performed, though the subjects predominantly exhibited ultrasound features consistent with severe-stage disease conditions. Third, the possibility of the presence of deep endometriotic nodules co-existing with adenomyosis could not be ruled out. Fourth, only cases of ovarian endometriosis were considered, limiting the assessment of changes in other endometriosis phenotypes. Fifth, the candidate genes and corresponding proteins identified as key regulators of ECM remodeling and fibrosis warrant histomorphological and immunohistochemical validation. These assessments could not be undertaken in the present study owing to the invasive nature of endometrial sampling, which limited tissue availability. Finally, the observations, though interesting, must be interpreted with caution since the analyzed datasets comprised a non-Asian population while the candidate genes were validated in an Indian patient cohort.

## Conclusion and future scope

For the first time, expression patterns of key genes and proteins in the eutopic endometrium of diffuse adenomyosis, ovarian endometriosis, co-existent adenomyosis–endometriosis, and healthy controls are compared. Most of the candidate genes could be associated with ECM remodeling and fibrotic changes, consistent with the pathogenic features of the disorders. These alterations in adenomyosis were indicative of an aggressive endo-myometrial environment. The IVF outcome parameters supported our observations. Further, MMP7, MMP9, and MMP11 appear promising as clinically relevant discriminatory markers of adenomyosis. The gene and protein expression pattern in the co-existent adenomyosis–endometriosis group was largely not consistent with adenomyosis or endometriosis. This tempts us to speculate that distinct disease-specific molecular responses could be triggered, which warrants further investigation of the co-occurring adenomyosis–endometriosis condition.

The present study provides a preliminary framework for understanding the molecular alterations associated with adenomyosis and endometriosis, highlighting directions to explore their translational relevance in clinical settings. Future studies on non-infertile populations and various subtypes and stages of the diseases are warranted to validate our observations. Investigations on additional MMPs and TIMPs, fibrosis-associated genes, pro-collagen genes, and downstream molecular pathways are required to deepen our understanding of the molecular mechanisms underlying these disorders.

## Supplementary Information

Supplementary material 1. Fig. S1. Workflow of the study design. The left panel in the image represents bioinformatic analysis of transcriptomic datasets on adenomyosis, endometriosis and controls. The right panel shows the patient recruitment criteria for validation of the bioinformatic findings. GEO: Gene Expression Omnibus, BMI: body mass index, MUSA: Morphological Uterus Sonographic Assessment, TVUS: transvaginal ultrasound, qRT-PCR: quantitative reverse transcription polymerase chain reaction. Fig. S2. Box plots for the adenomyosis and endometriosis datasets. Box plots demonstrate the distribution of normalized datasetsadenomyosis vs. healthy subjectsfrom GSE78851 datasetadenomyosis vs. healthy subjectsfrom GSE7307 dataset andendometriosis vs. healthy subjectsfrom the GSE7307 dataset. X and Y-axis represent the patients selected and their gene expression levels, respectively.Fig. S3. Representative transvaginal ultrasoundimages of patients.adenomyosis with asymmetric myometrial thickening, irregular endometrial–myometrial junctionmyometrial cystand heterogeneous myometriumendometriomaco-existent adenomyosis–endometriosis showing asymmetric myometrial wallscontrol with uniform endometrium–myometrium thickness Fig. S4. Full blots of the proteins of interest. The blots consist of four lanes showing four groups of patients: Adenomyosis- A, co-existent adenomyosis–endometriosis- AE, endometriosis- E and controls- C. The ladder showing the corresponding molecular weights are shown.-The blots showing the bands of target proteins and-showing the bands of beta-actin

## Data Availability

The data underlying this work are available in the article and its Supplementary material. The transcriptomic datasets are available in the GEO database (https://www.ncbi.nlm.nih.gov/geo/); Accession No: GSE78851 and GSE7307. The analysed differential expression data may be provided upon request.

## Abbreviations

DEGs: Differentially expressed genes
NGS: Next generation sequencing
ECM: Extracellular matrix
EMT: Epithelial–messenchymal transition
KRAS: Kirsten rat sarcoma viral oncogene homolog
qRT-PCR: Quantitative reverse transcription polymerase chain reaction
GEO: Gene expression omnibus
RMA: Robust multi-array average
GO: Gene Ontology
BP: Biological process
MF: Molecular function
CC: Cellular component
KEGG: Kyoto Encyclopedia of Genes and Genomes
FDR: False discovery rate
PPI: Protein–protein interaction network
STRING: Search tool for the retrieval of interacting genes/proteins
BMI: Body mass index
2D-TVUS: Two-dimensional transvaginal ultrasound sonography
MUSA: Morphological uterus sonographic assessment
BCA: Bicinchonic acid assay
TBST: Tris-buffered saline with Tween-20
SDS: Sodium dodecyl sulfate
HRP: Horseradish peroxidase
ECL: Enhanced chemiluminescence
ROC: Receiver operating characteristic
AUC: Area under the curve
E2: Estradiol
