# Supplementary material for: Bioinformatic analysis and validation of candidate genes in the eutopic endometrium reveal differential expressions in diffuse adenomyosis, endometrioma, and their co-existence
Source: Eur J Med Res. 2025 Nov 21;30:1154. doi: 10.1186/s40001-025-03412-7 (PMC12639679; doi:10.1186/s40001-025-03412-7)
Supplement: Supplementary file 1 — Supplementary material 1. Fig. S1. Workflow of the study design. The left panel in the image represents bioinformatic analysis of transcriptomic datasets on adenomyosis, endometriosis and controls. The right panel shows the patient recruitment criteria for validation of the bioinformatic findings. GEO: Gene Expression Omnibus, BMI: body mass index, MUSA: Morphological Uterus Sonographic Assessment, TVUS: transvaginal ultrasound, qRT-PCR: quantitative reverse transcription polymerase chain reaction. Fig. S2. Box plots for the adenomyosis and endometriosis datasets. Box plots demonstrate the distribution of normalized datasetsadenomyosis vs. healthy subjectsfrom GSE78851 datasetadenomyosis vs. healthy subjectsfrom GSE7307 dataset andendometriosis vs. healthy subjectsfrom the GSE7307 dataset. X and Y-axis represent the patients selected and their gene expression levels, respectively.Fig. S3. Representative transvaginal ultrasoundimages of patients.adenomyosis with asymmetric myometrial thickening, irregular endometrial–myometrial junctionmyometrial cystand heterogeneous myometriumendometriomaco-existent adenomyosis–endometriosis showing asymmetric myometrial wallscontrol with uniform endometrium–myometrium thickness Fig. S4. Full blots of the proteins of interest. The blots consist of four lanes showing four groups of patients: Adenomyosis- A, co-existent adenomyosis–endometriosis- AE, endometriosis- E and controls- C. The ladder showing the corresponding molecular weights are shown.-The blots showing the bands of target proteins and-showing the bands of beta-actin [file 40001_2025_3412_MOESM1_ESM.zip › New folder/Node scores_table S7.docx]

**Supplementary Table S7.** Node scores associated with MMP9 and TIMP1 in the network

| **Node 1** | **Node 2** | **Score** |
| --- | --- | --- |
| MMP9 | MMP11 | 0.920 |
| MMP9 | MMP7 | 0.963 |
| MMP9 | THBS1 | 0.970 |
| MMP9 | TIMP1 | 0.970 |
| MMP9 | SERPINA1 | 0.533 |
| MMP9 | IGFBP5 | 0.490 |
| TIMP1 | MMP11 | 0.920 |
| TIMP1 | MMP7 | 0.912 |
| TIMP1 | THBS1 | 0.520 |
| TIMP1 | IGFBP5 | <0.45 |
