# Supplementary material for: Bioinformatic analysis and validation of candidate genes in the eutopic endometrium reveal differential expressions in diffuse adenomyosis, endometrioma, and their co-existence
Source: Eur J Med Res. 2025 Nov 21;30:1154. doi: 10.1186/s40001-025-03412-7 (PMC12639679; doi:10.1186/s40001-025-03412-7)
Supplement: Supplementary file 1 — Supplementary material 1. Fig. S1. Workflow of the study design. The left panel in the image represents bioinformatic analysis of transcriptomic datasets on adenomyosis, endometriosis and controls. The right panel shows the patient recruitment criteria for validation of the bioinformatic findings. GEO: Gene Expression Omnibus, BMI: body mass index, MUSA: Morphological Uterus Sonographic Assessment, TVUS: transvaginal ultrasound, qRT-PCR: quantitative reverse transcription polymerase chain reaction. Fig. S2. Box plots for the adenomyosis and endometriosis datasets. Box plots demonstrate the distribution of normalized datasetsadenomyosis vs. healthy subjectsfrom GSE78851 datasetadenomyosis vs. healthy subjectsfrom GSE7307 dataset andendometriosis vs. healthy subjectsfrom the GSE7307 dataset. X and Y-axis represent the patients selected and their gene expression levels, respectively.Fig. S3. Representative transvaginal ultrasoundimages of patients.adenomyosis with asymmetric myometrial thickening, irregular endometrial–myometrial junctionmyometrial cystand heterogeneous myometriumendometriomaco-existent adenomyosis–endometriosis showing asymmetric myometrial wallscontrol with uniform endometrium–myometrium thickness Fig. S4. Full blots of the proteins of interest. The blots consist of four lanes showing four groups of patients: Adenomyosis- A, co-existent adenomyosis–endometriosis- AE, endometriosis- E and controls- C. The ladder showing the corresponding molecular weights are shown.-The blots showing the bands of target proteins and-showing the bands of beta-actin [file 40001_2025_3412_MOESM1_ESM.zip › New folder/R codes_online Supplementary file.docx]

**R codes used for analysis**

**GSE78851 (adenomyosis vs. controls; endometrium)**

# Set working directory

setwd("C:/Users/Admin/Downloads/AKS/ENDOMETRIOSIS/GSE7307/Adenomyosis/GSE78851/")

# Install and load necessary packages

if (!requireNamespace("BiocManager", quietly = TRUE))

install.packages("BiocManager")

BiocManager::install(c("GEOquery"))

BiocManager::install(c("limma"))

BiocManager::install("oligo")

library(oligo)

library(limma)

library(tidyverse)

library(GEOquery)

# Read and normalize the data using RMA

celFiles <- list.files('C:/Users/Admin/Downloads/AKS/ENDOMETRIOSIS/GSE7307/Adenomyosis/GSE78851/GSE78851_RAW/', full.name=TRUE)

raw.data <- read.celfiles(celFiles)

normalized.data <- rma(raw.data)

normalized.expr <- as.data.frame(exprs(normalized.data))

# Clean up column names

colnames(normalized.expr) <- gsub("_.*", "", colnames(normalized.expr))

eset <- normalized.expr

write.csv(eset, "GSE78851_normalized_expression.csv", row.names = FALSE)

# Create design matrix for differential expression analysis

design <- model.matrix(~factor(c(rep("disease", 3), rep("control", 5))))

colnames(design) <- c("Intercept", "Disease")

# Fit the linear model

fit <- lmFit(eset, design)

# Apply empirical Bayes statistics

fit <- eBayes(fit)

# Get differential expression results

results <- topTable(fit, coef="Disease", adjust.method="BH", number=Inf)

# Add gene symbols to results

gse <- getGEO("GSE78851", GSEMatrix = TRUE)

feature.data <- gse$GSE78851_series_matrix.txt.gz@featureData@data

feature.data <- feature.data[,c(1,10)]

colnames(results)

colnames(feature.data)

class(rownames(results))

results <- results %>%

rownames_to_column(var = 'ID') %>%

inner_join(feature.data %>% mutate(ID = as.character(ID)), by = 'ID')

colnames(results)[colnames(results) == "gene_assignment"] <- "Gene_Symbol"

results <- results %>%

mutate(Gene_Symbol = str_extract(Gene_Symbol, "(?<=//)[^/]+(?=//)"))

colnames(results)[colnames(results)=="Gene Symbol"] <- "Gene_Symbol"

# Order and export results

results <- results[order(results$adj.P.Val), ]

write.csv(results, "differential_expression_results.csv")

# Add regulation information

res_df <- as.data.frame(results) %>%

mutate(

significant = ifelse(adj.P.Val < 0.05 & abs(logFC) > 1, "Yes", "No"),

regulation = case_when(

adj.P.Val < 0.05 & logFC > 1 ~ "Up-regulated",

adj.P.Val < 0.05 & logFC < -1 ~ "Down-regulated",

TRUE ~ "Not-significant"

)

)

# Extract and export significantly differentially expressed genes

sig_genes <- res_df %>%

filter(adj.P.Val < 0.05 & abs(logFC) > 1)

write.csv(sig_genes, "GSE78851_FDEG_results.csv", row.names = FALSE)

# Step 13: Calculate and print summary statistics

total_genes <- nrow(res_df)

sig_up <- sum(res_df$regulation == "Up-regulated")

sig_down <- sum(res_df$regulation == "Down-regulated")

sig_total <- sig_up + sig_down

# Calculate and export summary statistics

summary_df <- data.frame(

Category = c("Up-regulated", "Down-regulated", "Not-significant"),

Count = c(sig_up, sig_down, total_genes - sig_total),

Percentage = c(sig_up/total_genes*100, sig_down/total_genes*100, (total_genes - sig_total)/total_genes*100)

)

summary_df$Percentage <- round(summary_df$Percentage, 2)

write.csv(summary_df, "regulation_summary.csv", row.names = FALSE)

# Create and save bar plot of regulation summary

ggplot(summary_df, aes(x = Category, y = Count, fill = Category)) +

geom_bar(stat = "identity") +

geom_text(aes(label = paste0(Count, " (", Percentage, "%)")),

position = position_stack(vjust = 0.5)) +

theme_minimal() +

labs(title = "Summary of Gene Regulation",

x = "Regulation Category",

y = "Number of Genes") +

scale_fill_manual(values = c("Up-regulated" = "red", "Down-regulated" = "green", "Not-significant" = "gray"))

ggsave("regulation_summary_plot.png", width = 10, height = 6, dpi = 300)

ggplot(summary_df %>% filter(Category != "Not-significant"),

aes(x = Category, y = Count, fill = Category)) +

geom_bar(stat = "identity") +

geom_text(aes(label = paste0(Count, " (", Percentage, "%)")),

position = position_stack(vjust = 0.5)) +

theme_minimal() +

labs(title = "Summary of Gene Regulation",

x = "Regulation Category",

y = "Number of Genes") +

scale_fill_manual(values = c("Up-regulated" = "red", "Down-regulated" = "green")) +

theme(legend.position = "none") # Removes legend since categories are on x-axis

ggsave("regulation_summary_plot_2.png", width = 10, height = 6, dpi = 300)

# Create and save volcano plot

logFC_threshold <- 1

adj.pvalue_threshold <- 0.05

res_df$regulation <- factor(res_df$regulation,

levels = c("Up-regulated", "Down-regulated", "Not-significant"))

vplot <- ggplot(res_df, aes(x = logFC, y = -log10(P.Value))) +

geom_point(aes(color = regulation), alpha = 0.8, size = 3) +

scale_color_manual(

values = c("Up-regulated" = "red",

"Down-regulated" = "blue",

"Not-significant" = "gray"),

breaks = c("Up-regulated", "Down-regulated", "Not-significant"),

labels = c("Up-regulated", "Down-regulated", "Not-significant"),

drop = FALSE

) +

theme_minimal() +

labs(

title = "Volcano Plot",

x = "Log2Fold Change (logFC)",

y = "-Log10 P-value",

color = "Gene Expression"

) +

theme(

plot.title = element_text(hjust = 0.5, size = 16), # Increased title font size

axis.title.x = element_text(size = 14), # Increased x-axis title size

axis.title.y = element_text(size = 14), # Increased y-axis title size

axis.text.x = element_text(size = 12), # Increased x-axis tick labels size

axis.text.y = element_text(size = 12), # Increased y-axis tick labels size

legend.title = element_text(size = 14), # Increased legend title size

legend.text = element_text(size = 12) # Increased legend text size

) +

geom_hline(yintercept = -log10(adj.pvalue_threshold), linetype = "dashed", color = "black") +

geom_vline(xintercept = c(-logFC_threshold, logFC_threshold), linetype = "dashed", color = "black")

# Print the plot

print(vplot)

ggsave("volcano_plot.png", vplot, width = 12, height = 10, dpi = 300)

**GSE7307 (adenomyosis vs. controls; myometrium)**

# Set working directory

setwd("C:/Users/Admin/Downloads/AKS/ENDOMETRIOSIS/GSE7307/Adenomyosis/GSE7307_MYOMETRIUM/")

# Install and load necessary packages

if (!requireNamespace("BiocManager", quietly = TRUE))

install.packages("BiocManager")

BiocManager::install(c("affy", "limma"))

library(affy)

library(limma)

library(tidyverse)

library(GEOquery)

# Specify the directory containing raw data files

directory <- "C:/Users/Admin/Downloads/AKS/ENDOMETRIOSIS/GSE7307/GSE7307_RAW"

# Read and normalize the data

raw.data <- ReadAffy(celfile.path = directory)

normalized.data <- affy::rma(raw.data)

normalized.expr <- as.data.frame(exprs(normalized.data))

# Clean up column names

colnames(normalized.expr) <- gsub("\\.CEL\\.gz$", "", colnames(normalized.expr))

normalized.expr <- normalized.expr[, c("GSM176242", "GSM176244", "GSM176246", "GSM176248", "GSM176250",

"GSM176252", "GSM176255", "GSM176257", "GSM176259", "GSM176261",

"GSM176102", "GSM176104", "GSM176106", "GSM176108", "GSM176110",

"GSM176126", "GSM176128", "GSM176133", "GSM176138", "GSM176228")]

eset <- normalized.expr

write.csv(eset, "GSE7307_Adenomyosis_normalized_expression.csv", row.names = FALSE)

# Create design matrix for differential expression analysis

design <- model.matrix(~factor(c(rep("disease", 10), rep("control", 10))))

colnames(design) <- c("Intercept", "Disease")

# Fit the linear model

fit <- lmFit(eset, design)

# Apply empirical Bayes statistics

fit <- eBayes(fit)

# Get differential expression results

results <- topTable(fit, coef="Disease", adjust.method="BH", number=Inf)

# Add gene symbols to results

gse <- getGEO("GSE7307", GSEMatrix = TRUE)

feature.data <- gse$GSE7307_series_matrix.txt.gz@featureData@data

feature.data <- feature.data[,c(1,11)]

results <- results %>%

rownames_to_column(var = 'ID') %>%

inner_join(feature.data %>% mutate(ID = as.character(ID)), by = 'ID')

# Order and export results

results <- results[order(results$adj.P.Val), ]

write.csv(results, "differential_expression_results.csv")

colnames(results)[colnames(results)=="Gene Symbol"] <- "Gene_Symbol"

# Add regulation information

res_df <- as.data.frame(results) %>%

mutate(

significant = ifelse(adj.P.Val < 0.05 & abs(logFC) > 1, "Yes", "No"),

regulation = case_when(

adj.P.Val < 0.05 & logFC > 1 ~ "Up-regulated",

adj.P.Val < 0.05 & logFC < -1 ~ "Down-regulated",

TRUE ~ "Not-significant"

)

)

# Extract and export significantly differentially expressed genes

sig_genes <- res_df %>%

filter(adj.P.Val < 0.05 & abs(logFC) > 1)

write.csv(sig_genes, "GSE7307_Adenomyosis_FDEG_results.csv", row.names = FALSE)

# Calculate and export summary statistics

total_genes <- nrow(res_df)

sig_up <- sum(res_df$regulation == "Up-regulated")

sig_down <- sum(res_df$regulation == "Down-regulated")

sig_total <- sig_up + sig_down

summary_df <- data.frame(

Category = c("Up-regulated", "Down-regulated", "Not-significant"),

Count = c(sig_up, sig_down, total_genes - sig_total),

Percentage = c(sig_up/total_genes*100, sig_down/total_genes*100, (total_genes - sig_total)/total_genes*100)

)

summary_df$Percentage <- round(summary_df$Percentage, 2)

write.csv(summary_df, "regulation_summary.csv", row.names = FALSE)

# Create and save bar plot of regulation summary

ggplot(summary_df, aes(x = Category, y = Count, fill = Category)) +

geom_bar(stat = "identity") +

geom_text(aes(label = paste0(Count, " (", Percentage, "%)")),

position = position_stack(vjust = 0.5)) +

theme_minimal() +

labs(title = "Summary of Gene Regulation",

x = "Regulation Category",

y = "Number of Genes") +

scale_fill_manual(values = c("Up-regulated" = "red", "Down-regulated" = "green", "Not-significant" = "gray"))

ggsave("regulation_summary_plot.png", width = 10, height = 6, dpi = 300)

ggplot(summary_df %>% filter(Category != "Not-significant"),

aes(x = Category, y = Count, fill = Category)) +

geom_bar(stat = "identity") +

geom_text(aes(label = paste0(Count, " (", Percentage, "%)")),

position = position_stack(vjust = 0.5)) +

theme_minimal() +

labs(title = "Summary of Gene Regulation",

x = "Regulation Category",

y = "Number of Genes") +

scale_fill_manual(values = c("Up-regulated" = "red", "Down-regulated" = "green")) +

theme(legend.position = "none") # Removes legend since categories are on x-axis

ggsave("regulation_summary_plot_2.png", width = 10, height = 6, dpi = 300)

# Create and save volcano plot

logFC_threshold <- 1

adj.pvalue_threshold <- 0.05

res_df$regulation <- factor(res_df$regulation,

levels = c("Up-regulated", "Down-regulated", "Not-significant"))

vplot <- ggplot(res_df, aes(x = logFC, y = -log10(P.Value))) +

geom_point(aes(color = regulation), alpha = 0.8, size = 3) +

scale_color_manual(

values = c("Up-regulated" = "red",

"Down-regulated" = "blue",

"Not-significant" = "gray"),

breaks = c("Up-regulated", "Down-regulated", "Not-significant"),

labels = c("Up-regulated", "Down-regulated", "Not-significant"),

drop = FALSE

) +

theme_minimal() +

labs(

title = "Volcano Plot",

x = "Log2Fold Change (logFC)",

y = "-Log10 P-value",

color = "Gene Expression"

) +

theme(

plot.title = element_text(hjust = 0.5, size = 16), # Increased title font size

axis.title.x = element_text(size = 14), # Increased x-axis title size

axis.title.y = element_text(size = 14), # Increased y-axis title size

axis.text.x = element_text(size = 12), # Increased x-axis tick labels size

axis.text.y = element_text(size = 12), # Increased y-axis tick labels size

legend.title = element_text(size = 14), # Increased legend title size

legend.text = element_text(size = 12) # Increased legend text size

) +

geom_hline(yintercept = -log10(adj.pvalue_threshold), linetype = "dashed", color = "black") +

geom_vline(xintercept = c(-logFC_threshold, logFC_threshold), linetype = "dashed", color = "black")

# Print the plot

print(vplot)

ggsave("volcano_plot.png", vplot, width = 12, height = 10, dpi = 600)

**GSE7307 (endometriosis vs. controls)**

# Set working directory

setwd("C:/Users/Admin/Downloads/AKS/ENDOMETRIOSIS/GSE7307/Endometriosis/")

# Install and load necessary packages

if (!requireNamespace("BiocManager", quietly = TRUE))

install.packages("BiocManager")

BiocManager::install(c("affy", "limma"))

library(affy)

library(limma)

library(tidyverse)

library(GEOquery)

# Specify the directory containing raw data files

directory <- "C:/Users/Admin/Downloads/AKS/ENDOMETRIOSIS/GSE7307/GSE7307_RAW"

# Read and normalize the data

raw.data <- ReadAffy(celfile.path = directory)

normalized.data <- affy::rma(raw.data)

normalized.expr <- as.data.frame(exprs(normalized.data))

# Clean up column names

colnames(normalized.expr) <- gsub("\\.CEL\\.gz$", "", colnames(normalized.expr))

normalized.expr <- normalized.expr[, c("GSM175786", "GSM175787", "GSM175788", "GSM176082", "GSM176083",

"GSM176084", "GSM176085", "GSM176086", "GSM176087", "GSM176088",

"GSM176089", "GSM176090", "GSM176091", "GSM176092", "GSM176234",

"GSM176236", "GSM176238", "GSM176240", "GSM176039", "GSM176041",

"GSM176043", "GSM176093", "GSM176094", "GSM176095", "GSM176096",

"GSM176097", "GSM176098", "GSM176099", "GSM176100", "GSM176101",

"GSM176127", "GSM176132", "GSM176137", "GSM176141", "GSM176142",

"GSM176319")]

eset <- normalized.expr

write.csv(eset, "GSE7307_normalized_expression.csv", row.names = FALSE)

# Create design matrix for differential expression analysis

design <- model.matrix(~factor(c(rep("disease", 18), rep("control", 18))))

colnames(design) <- c("Intercept", "Disease")

# Fit the linear model

fit <- lmFit(eset, design)

# Apply empirical Bayes statistics

fit <- eBayes(fit)

# Get differential expression results

results <- topTable(fit, coef="Disease", adjust.method="BH", number=Inf)

# Add gene symbols to results

gse <- getGEO("GSE7307", GSEMatrix = TRUE)

feature.data <- gse$GSE7307_series_matrix.txt.gz@featureData@data

feature.data <- feature.data[,c(1,11)]

results <- results %>%

rownames_to_column(var = 'ID') %>%

inner_join(feature.data %>% mutate(ID = as.character(ID)), by = 'ID')

# Order and export results

results <- results[order(results$adj.P.Val), ]

write.csv(results, "differential_expression_results_new.csv")

colnames(results)[colnames(results)=="Gene Symbol"] <- "Gene_Symbol"

# Add regulation information

res_df <- as.data.frame(results) %>%

mutate(

significant = ifelse(adj.P.Val < 0.05 & abs(logFC) > 1, "Yes", "No"),

regulation = case_when(

adj.P.Val < 0.05 & logFC > 1 ~ "Up-regulated",

adj.P.Val < 0.05 & logFC < -1 ~ "Down-regulated",

TRUE ~ "Not-significant"

)

)

# Extract and export significantly differentially expressed genes

sig_genes <- res_df %>%

filter(adj.P.Val < 0.05 & abs(logFC) > 1)

write.csv(sig_genes, "GSE7307_FDEG_results.csv", row.names = FALSE)

# Calculate and export summary statistics

total_genes <- nrow(res_df)

sig_up <- sum(res_df$regulation == "Up-regulated")

sig_down <- sum(res_df$regulation == "Down-regulated")

sig_total <- sig_up + sig_down

summary_df <- data.frame(

Category = c("Up-regulated", "Down-regulated", "Not-significant"),

Count = c(sig_up, sig_down, total_genes - sig_total),

Percentage = c(sig_up/total_genes*100, sig_down/total_genes*100, (total_genes - sig_total)/total_genes*100)

)

summary_df$Percentage <- round(summary_df$Percentage, 2)

write.csv(summary_df, "regulation_summary.csv", row.names = FALSE)

# Create and save bar plot of regulation summary

ggplot(summary_df, aes(x = Category, y = Count, fill = Category)) +

geom_bar(stat = "identity") +

geom_text(aes(label = paste0(Count, " (", Percentage, "%)")),

position = position_stack(vjust = 0.5)) +

theme_minimal() +

labs(title = "Summary of Gene Regulation",

x = "Regulation Category",

y = "Number of Genes") +

scale_fill_manual(values = c("Up-regulated" = "red", "Down-regulated" = "green", "Not-significant" = "gray"))

ggsave("regulation_summary_plot.png", width = 10, height = 6, dpi = 300)

ggplot(summary_df %>% filter(Category != "Not-significant"),

aes(x = Category, y = Count, fill = Category)) +

geom_bar(stat = "identity") +

geom_text(aes(label = paste0(Count, " (", Percentage, "%)")),

position = position_stack(vjust = 0.5)) +

theme_minimal() +

labs(title = "Summary of Gene Regulation",

x = "Regulation Category",

y = "Number of Genes") +

scale_fill_manual(values = c("Up-regulated" = "red", "Down-regulated" = "green")) +

theme(legend.position = "none") # Removes legend since categories are on x-axis

ggsave("regulation_summary_plot_2.png", width = 10, height = 6, dpi = 300)

# Create and save volcano plot

logFC_threshold <- 1

adj.pvalue_threshold <- 0.05

res_df$regulation <- factor(res_df$regulation,

levels = c("Up-regulated", "Down-regulated", "Not-significant"))

vplot <- ggplot(res_df, aes(x = logFC, y = -log10(P.Value))) +

geom_point(aes(color = regulation), alpha = 0.8, size = 3) +

scale_color_manual(

values = c("Up-regulated" = "red",

"Down-regulated" = "blue",

"Not-significant" = "gray"),

breaks = c("Up-regulated", "Down-regulated", "Not-significant"),

labels = c("Up-regulated", "Down-regulated", "Not-significant"),

drop = FALSE

) +

theme_minimal() +

labs(

title = "Volcano Plot",

x = "Log2Fold Change (logFC)",

y = "-Log10 P-value",

color = "Gene Expression"

) +

theme(

plot.title = element_text(hjust = 0.5, size = 16), # Increased title font size

axis.title.x = element_text(size = 14), # Increased x-axis title size

axis.title.y = element_text(size = 14), # Increased y-axis title size

axis.text.x = element_text(size = 12), # Increased x-axis tick labels size

axis.text.y = element_text(size = 12), # Increased y-axis tick labels size

legend.title = element_text(size = 14), # Increased legend title size

legend.text = element_text(size = 12) # Increased legend text size

) +

geom_hline(yintercept = -log10(adj.pvalue_threshold), linetype = "dashed", color = "black") +

geom_vline(xintercept = c(-logFC_threshold, logFC_threshold), linetype = "dashed", color = "black")

# Print the plot

print(vplot)

ggsave("volcano_plot.png", vplot, width = 12, height = 10, dpi = 600)
