# Supplementary material for: Bioinformatic analysis and validation of candidate genes in the eutopic endometrium reveal differential expressions in diffuse adenomyosis, endometrioma, and their co-existence
Source: Eur J Med Res. 2025 Nov 21;30:1154. doi: 10.1186/s40001-025-03412-7 (PMC12639679; doi:10.1186/s40001-025-03412-7)
Supplement: Supplementary file 1 — Supplementary material 1. Fig. S1. Workflow of the study design. The left panel in the image represents bioinformatic analysis of transcriptomic datasets on adenomyosis, endometriosis and controls. The right panel shows the patient recruitment criteria for validation of the bioinformatic findings. GEO: Gene Expression Omnibus, BMI: body mass index, MUSA: Morphological Uterus Sonographic Assessment, TVUS: transvaginal ultrasound, qRT-PCR: quantitative reverse transcription polymerase chain reaction. Fig. S2. Box plots for the adenomyosis and endometriosis datasets. Box plots demonstrate the distribution of normalized datasetsadenomyosis vs. healthy subjectsfrom GSE78851 datasetadenomyosis vs. healthy subjectsfrom GSE7307 dataset andendometriosis vs. healthy subjectsfrom the GSE7307 dataset. X and Y-axis represent the patients selected and their gene expression levels, respectively.Fig. S3. Representative transvaginal ultrasoundimages of patients.adenomyosis with asymmetric myometrial thickening, irregular endometrial–myometrial junctionmyometrial cystand heterogeneous myometriumendometriomaco-existent adenomyosis–endometriosis showing asymmetric myometrial wallscontrol with uniform endometrium–myometrium thickness Fig. S4. Full blots of the proteins of interest. The blots consist of four lanes showing four groups of patients: Adenomyosis- A, co-existent adenomyosis–endometriosis- AE, endometriosis- E and controls- C. The ladder showing the corresponding molecular weights are shown.-The blots showing the bands of target proteins and-showing the bands of beta-actin [file 40001_2025_3412_MOESM1_ESM.zip › New folder/Supplementary Table S4.docx]

**Supplementary Table S4**. Gene Ontology (GO) terms associated with the common differentially expressed genes (DEGs)

| **GO:ID** | **Name** | **Proteins involved** | **P value** | **Adj. p value** |
| --- | --- | --- | --- | --- |
| **Biological processes** | | | | |
| GO:0017015 | Regulation of transforming growth factor Beta receptor signaling pathway | MECOM, NREP, THBS1 | 0.0003 | 0.06 |
| GO:0050920 | Regulation of chemotaxis | EFNB2, THBS1 | 0.0005 | 0.06 |
| GO:0030574 | Collagen catabolic process | MMP7, MMP11 | 0.0005 | 0.06 |
| GO:0030336 | Negative regulation of cell migration | MMP7, IGFBP5, ZMYND8, THBS1 | 0.001 | 0.06 |
| GO:0022617 | Extracellular matrix disassembly | MMP11, MMP7 | 0.001 | 0.06 |
| GO:0022411 | Cellular component disassembly | MMP11, MMP7 | 0.001 | 0.06 |
| GO:0048661 | Positive regulation of smooth muscle cell proliferation | IGFBP5, THBS1 | 0.001 | 0.06 |
| GO:0006417 | Regulation of translation | IGFBP5, ENC1, THBS1 | 0.001 | 0.06 |
| GO:0048660 | Regulation of smooth muscle cell proliferation | IGFBP5, THBS1 | 0.001 | 0.06 |
| GO:0030334 | Regulation of cell migration | MMP11, ENC1, THBS1 | 0.002 | 0.06 |
| **Molecular functions** | | | | |
| GO:0004252 | Serine-type endopeptidase activity | MMP11, MMP7, PCSK5 | 0.0003 | 0.018 |
| GO:0008236 | Serine-type peptidase activity | MMP11, MMP7, PCSK5 | 0.0005 | 0.018 |
| GO:0004175 | Endopeptidase activity | MMP11, MMP7, PCSK5 | 0.006 | 0.06 |
| GO:0004222 | Metallo-endopeptidase activity | MMP11, MMP7 | 0.005 | 0.06 |
| GO:0016723 | Oxidoreductase activity, Acting on metal ions, NAD or NADP as acceptor | STEAP4 | 0.005 | 0.05 |
| GO:0031995 | Insulin-like growth factor II binding | IGFBP5 | 0.006 | 0.06 |
| GO:0002020 | Protease binding | SERPINA1, THBS1 | 0.0089 | 0.06 |
| GO:0004866 | Endopeptidase inhibitor activity | SERPINA1, THBS1 | 0.007 | 0.06 |
| GO:0008237 | Metallopeptidase activity | MMP11, MMP7 | 0.008 | 0.06 |
| GO:0030292 | Protein tyrosine kinase inhibitor activity | PTPRC | 0.009 | 0.05 |
| **Cellular components** | | | | |
| GO:0031093 | Platelet alpha granule lumen | SERPINA1, THBS1 | 0.0025 | 0.041 |
| GO:0070013 | Intracellular organelle lumen | MMP11, SERPINA1, IGFBP5, PCSK5, THBS1 | 0.0025 | 0.041 |
| GO:0005788 | Endoplasmic reticulum lumen | SERPINA1, IGFBP5, THBS1 | 0.004 | 0.041 |
| GO:0031091 | Platelet alpha granule | SERPINA1, THBS1 | 0.004 | 0.041 |
| GO:0005796 | Golgi lumen | MMP11, PCSK5 | 0.005 | 0.042 |
| GO:0005859 | Muscle myosin complex | MYH11 | 0.015 | 0.088 |
| GO:0045121 | Membrane raft | PTPRC, SLC39A6 | 0.016 | 0.081 |
| GO:0098588 | Bounding membrane of organelle | SERPINA1, STEAP4, PTPRC, PCSK5 | 0.016 | 0.081 |
| GO:0032982 | Myosin filament | MYH11 | 0.018 | 0.081 |
| GO:0099512 | Supramolecular fiber | MYH11 | 0.04 | 0.15 |
| Pathways associated with common differentially expressed genes (DEGs) | | | | |
| **Reactome pathways** | | | | |
| **Name** | **Reactome ID** | **Proteins involved** | **P value** | **Adj. p value** |
| Activation of matrix metalloproteinases | R-HSA-1592389 | MMP11, MMP7 | 0.0006 | 0.06 |
| Collagen degradation | R-HSA-1442490 | MMP11, MMP7 | 0.002 | 0.08 |
| Semaphorin interactions | R-HSA-373755 | PTPRC, MYH11 | 0.002 | 0.08 |
| Extracellular matrix organization | R-HSA-1474244 | MMP11, MMP7, THBS1 | 0.004 | 0.09 |
| Eph-ephrin signaling | R-HSA-2682334 | EFNB2, MYH11 | 0.004 | 0.09 |
| Post-translational protein phosphorylation | R-HSA-8957275 | SERPINA1, IGFBP5 | 0.006 | 0.09 |
| Regulation of IGF transport and uptake by insulin-like growth factor binding proteins (IGFBPs) | R-HSA-381426 | SERPINA1, IGFBP5 | 0.008 | 0.09 |
| Platelet degranulation | R-HSA-114608 | SERPINA1, IGFBP5 | 0.01 | 0.09 |
| Response to elevated platelet cytosolic Ca2+ | R-HSA-76005 | SERPINA1, IGFBP5 | 0.01 | 0.09 |
| Degradation of the extracellular matrix | R-GGA-1474228 | MMP11, MMP7 | 0.009 | 0.09 |
| **KEGG pathways** | | | | |
| **Name** | **KEGG ID** | **Proteins involved** | **P value** | **Adj. p value** |
| Proteoglycans in cancer | hsa05205 | THBS1, HOXD10 | 0.02 | 0.27 |
| Primary immunodeficiency | hsa05340 | PTPRC | 0.04 | 0.27 |
| Bladder cancer | hsa05219 | THBS1 | 0.04 | 0.27 |
| MicroRNAs in cancer | hsa05206 | THBS1, HOXD10 | 0.04 | 0.27 |
| Malaria | hsa05144 | THBS1 | 0.05 | 0.27 |
| Lysine degradation | hsa00310 | MECOM | 0.07 | 0.27 |
| p53 signaling pathway | hsa04115 | THBS1 | 0.08 | 0.27 |
| Chronic myeloid leukemia | hsa05220 | MECOM | 0.08 | 0.27 |
| Complement and coagulation cascades | hsa04610 | SERPINA1 | 0.09 | 0.27 |
| ECM-receptor interaction | hsa04512 | THBS1 | 0.09 | 0.27 |

Adj. p value<0.05 was considered as statistically significant
